# Supplementary figures and images for: Enhancing lobaplatin sensitivity in lung adenocarcinoma through inhibiting LDHA-targeted metabolic pathways
Source: PLoS One. 2024 Dec 16;19(12):e0310825. doi: 10.1371/journal.pone.0310825 (PMC11649076; doi:10.1371/journal.pone.0310825)

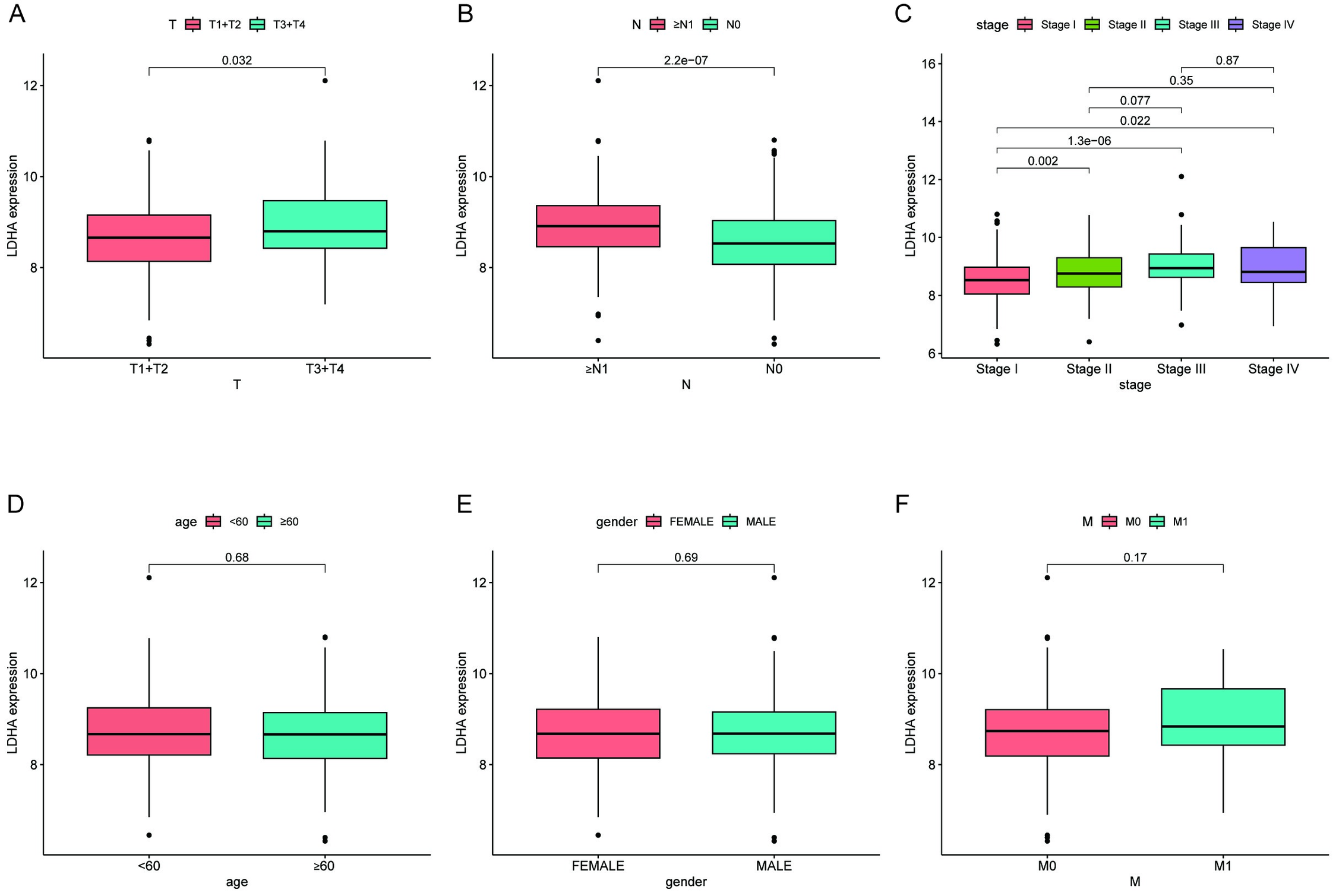

Supplement: S1 Fig — (A) T1+T2 vs. T3+T4. (B) N0 vs. ≥N1 (N1+N2). (C) Comparison among four levels. (D) Under 60 years old vs. ≥60 years old. (E) Female vs. male. (F) M0 vs. M1. (TIF) [file pone.0310825.s001.tif]

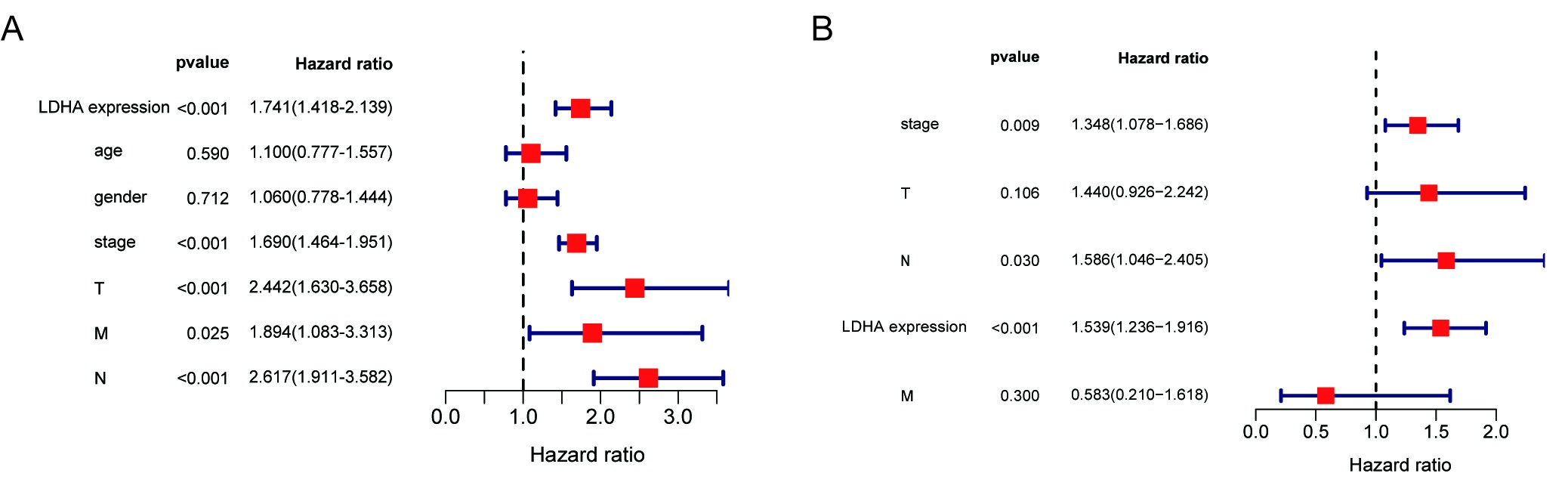

Supplement: S2 Fig — (A) Univariate Cox regression analysis of overall survival with LDHA expression and clinical characteristics. (B) Multivariate Cox regression analysis of overall survival with LDHA expression and clinical characteristics. (TIF) [file pone.0310825.s002.tif]
