# Supplementary material for: Enhancing lobaplatin sensitivity in lung adenocarcinoma through inhibiting LDHA-targeted metabolic pathways
Source: PLoS One. 2024 Dec 16;19(12):e0310825. doi: 10.1371/journal.pone.0310825 (PMC11649076; doi:10.1371/journal.pone.0310825)
Supplement: S1 Table — (DOCX) [file pone.0310825.s003.docx]

Table S1 KEGG enrichment results of differentially expressed genes between low and low LDHA expression groups

| ID | Description | GeneRatio | BgRatio | pvalue | p.adjust | qvalue | geneID |
| --- | --- | --- | --- | --- | --- | --- | --- |
| hsa04151 | PI3K-Akt signaling pathway | 25/254 | 359/8644 | ≤0.001 | 0.002 | 0.002 | NRAS/SPP1/CCNE1/BRCA1/TNXB/ITGAV/ITGA8/LAMC2/OSMR/TNC/ITGA5/COL4A3/IBSP/VEGFC/IL6R/TGFA/COL4A4/FGFR4/FGFR3/COL9A2/ITGA2/LAMB3/THBS2/COL1A1/AREG |
| hsa05165 | Human papillomavirus infection | 20/254 | 331/8644 | 0.002 | 0.028 | 0.024 | PKM/CCNA2/NRAS/SPP1/CCNE1/TNXB/ITGAV/ITGA8/TUBG1/LAMC2/TNC/ITGA5/COL4A3/IBSP/COL4A4/COL9A2/ITGA2/LAMB3/THBS2/COL1A1 |
| hsa04510 | Focal adhesion | 17/254 | 203/8644 | ≤0.001 | 0.003 | 0.002 | SPP1/TNXB/ITGAV/ITGA8/LAMC2/TNC/ITGA5/COL4A3/IBSP/VEGFC/COL4A4/COL9A2/ITGA2/LAMB3/RASGRF1/THBS2/COL1A1 |
| hsa04110 | Cell cycle | 19/254 | 157/8644 | ≤0.001 | ≤0.001 | ≤0.001 | CCNB1/MAD2L1/CCNA2/CDK1/CDC6/MCM4/MCM6/CDC25C/ORC1/CDC20/PLK1/TTK/CCNE1/TRIP13/CDC25A/MCM2/PKMYT1/ESPL1/TFDP1 |
| hsa01200 | Carbon metabolism | 10/254 | 115/8644 | 0.002 | 0.03 | 0.026 | PGK1/GAPDH/PGAM1/PKM/ENO1/PFKP/HK2/ENO2/ALDOC/RGN |
| hsa04066 | HIF-1 signaling pathway | 12/254 | 109/8644 | ≤0.001 | 0.002 | 0.002 | LDHA/PGK1/GAPDH/ENO1/SLC2A1/HIF1A/PFKP/HK2/ENO2/ALDOC/IL6R/SERPINE1 |
| hsa04974 | Protein digestion and absorption | 9/254 | 103/8644 | 0.003 | 0.044 | 0.038 | ATP1A2/ATP1B2/ELN/COL11A1/COL4A3/COL14A1/COL4A4/COL9A2/COL1A1 |
| hsa04657 | IL-17 signaling pathway | 13/254 | 94/8644 | ≤0.001 | ≤0.001 | ≤0.001 | CCL7/FOSL1/S100A9/CXCL8/MMP1/S100A8/CCL20/MMP3/CXCL10/FOSB/S100A7/MMP13/CXCL5 |
| hsa05222 | Small cell lung cancer | 9/254 | 92/8644 | ≤0.001 | 0.026 | 0.023 | CKS2/CKS1B/CCNE1/ITGAV/LAMC2/COL4A3/COL4A4/ITGA2/LAMB3 |
| hsa04512 | ECM-receptor interaction | 16/254 | 89/8644 | ≤0.001 | ≤0.001 | ≤0.001 | HMMR/SPP1/TNXB/ITGAV/ITGA8/LAMC2/TNC/ITGA5/COL4A3/IBSP/COL4A4/COL9A2/ITGA2/LAMB3/THBS2/COL1A1 |
| hsa04610 | Complement and coagulation cascades | 13/254 | 86/8644 | ≤0.001 | ≤0.001 | ≤0.001 | C7/PLAU/F10/PLAUR/CLU/A2M/F12/C6/C2/SERPINE1/C4BPA/CR2/C5 |
| hsa01232 | Nucleotide metabolism | 11/254 | 85/8644 | ≤0.001 | 0.002 | 0.001 | RRM2/HPRT1/TK1/RRM1/NME1/TYMS/CTPS1/XDH/CDA/UCK2/NT5E |
| hsa05412 | Arrhythmogenic right ventricular cardiomyopathy | 10/254 | 84/8644 | ≤0.001 | 0.004 | 0.004 | DSG2/DES/ITGAV/ITGA8/DSC2/ITGA5/SGCA/GJA1/PKP2/ITGA2 |
| hsa00983 | Drug metabolism - other enzymes | 8/254 | 80/8644 | 0.002 | 0.033 | 0.029 | RRM2/HPRT1/TK1/RRM1/NME1/XDH/CDA/UCK2 |
| hsa01230 | Biosynthesis of amino acids | 8/254 | 75/8644 | 0.002 | 0.026 | 0.023 | PGK1/GAPDH/PGAM1/PKM/ENO1/PFKP/ENO2/ALDOC |
| hsa04115 | p53 signaling pathway | 8/254 | 74/8644 | 0.002 | 0.026 | 0.023 | CCNB1/RRM2/CDK1/PMAIP1/CCNE1/IGFBP3/SERPINE1/SERPINB5 |
| hsa05230 | Central carbon metabolism in cancer | 9/254 | 70/8644 | ≤0.001 | 0.004 | 0.004 | LDHA/PGAM1/PKM/SLC2A1/NRAS/HIF1A/PFKP/HK2/FGFR3 |
| hsa00010 | Glycolysis / Gluconeogenesis | 12/254 | 67/8644 | ≤0.001 | ≤0.001 | ≤0.001 | LDHA/PGK1/GAPDH/PGAM1/PKM/ENO1/ADH1B/PFKP/ALDH1B1/HK2/ENO2/ALDOC |
| hsa00240 | Pyrimidine metabolism | 9/254 | 58/8644 | ≤0.001 | 0.002 | 0.001 | RRM2/TK1/RRM1/NME1/TYMS/CTPS1/CDA/UCK2/NT5E |
| hsa03030 | DNA replication | 6/254 | 36/8644 | ≤0.001 | 0.012 | 0.01 | MCM4/MCM6/PRIM1/RFC3/MCM2/RFC4 |
