# Supplementary material for: Enhancing lobaplatin sensitivity in lung adenocarcinoma through inhibiting LDHA-targeted metabolic pathways
Source: PLoS One. 2024 Dec 16;19(12):e0310825. doi: 10.1371/journal.pone.0310825 (PMC11649076; doi:10.1371/journal.pone.0310825)
Supplement: S1 Raw images — (PDF) [file pone.0310825.s004.pdf]

Fig 1C

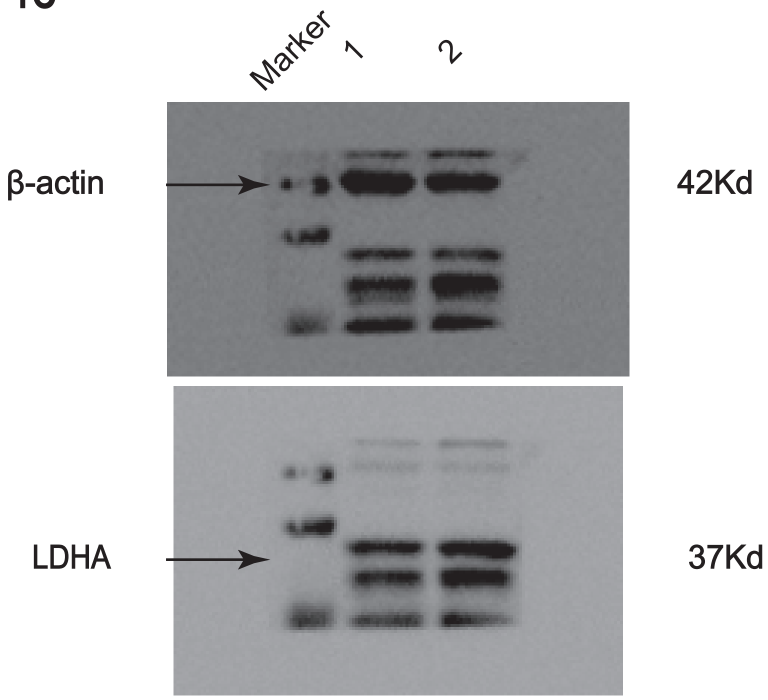

Figure 1 . Fluorescent imaging. 1. Paracancerous, 2. Lung adenocarcinoma tissue

Fig 2A

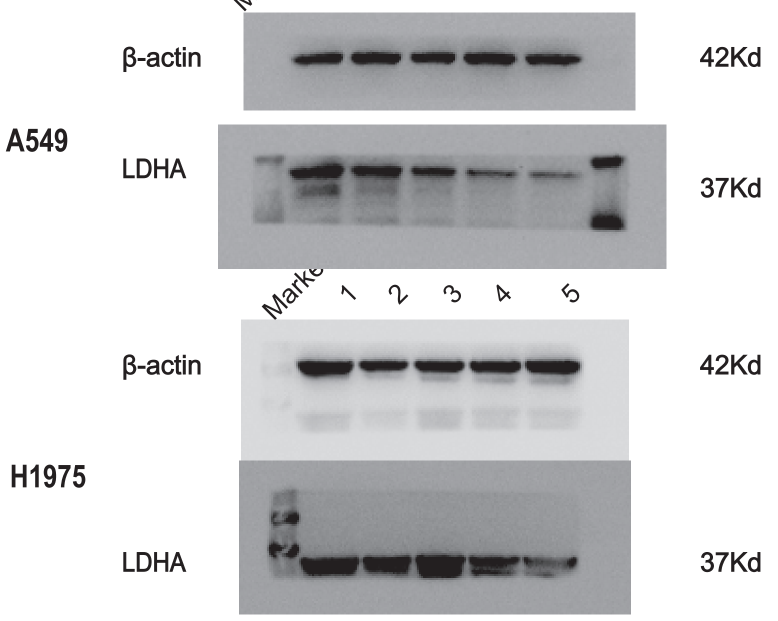

Figure 2A． Fluorescent imaging. 1. Con, 2. si-NC, 3. si-1, 4. si-2, 5.si-3

Fig 2E

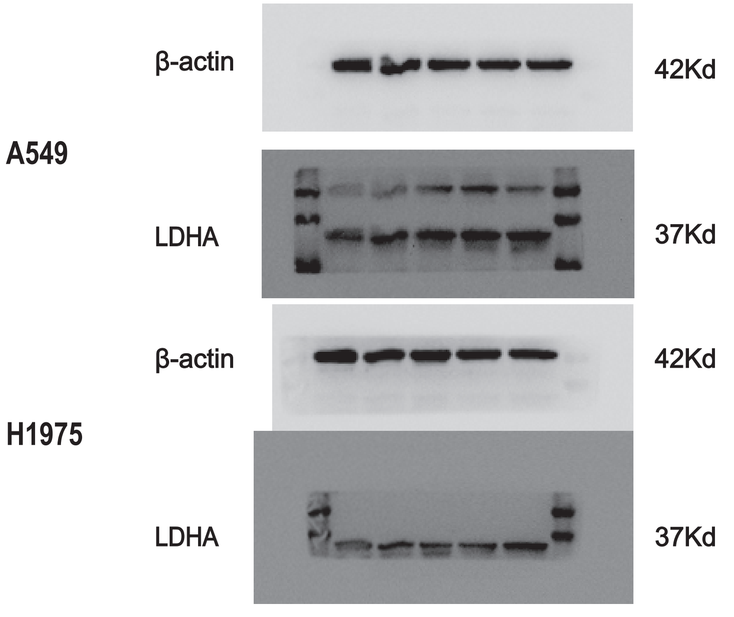

Figure 2E． Fluorescent imaging. 1. Con, 2. 24h, 3. 48h, 4. 72h, 5.96h

Fig 3B

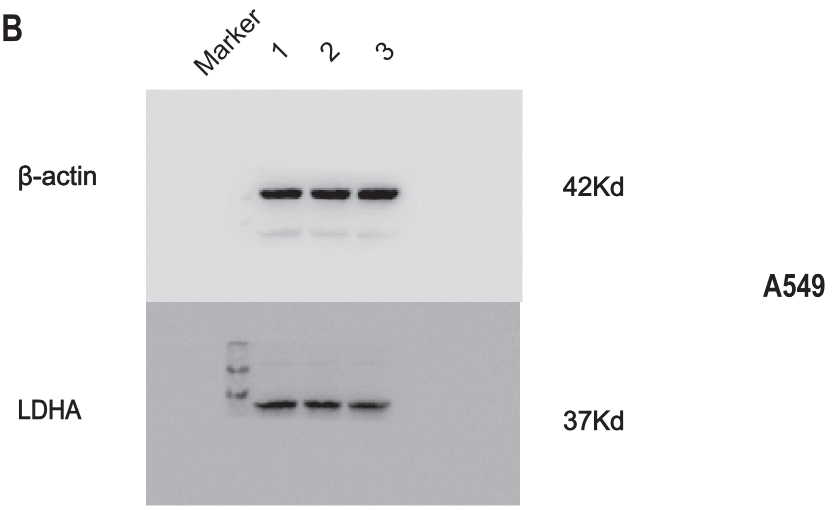

Figure 3B(A549). Fluorescent imaging. 1. Con, 2. 20nM, 3. 40nM(Oxmate)

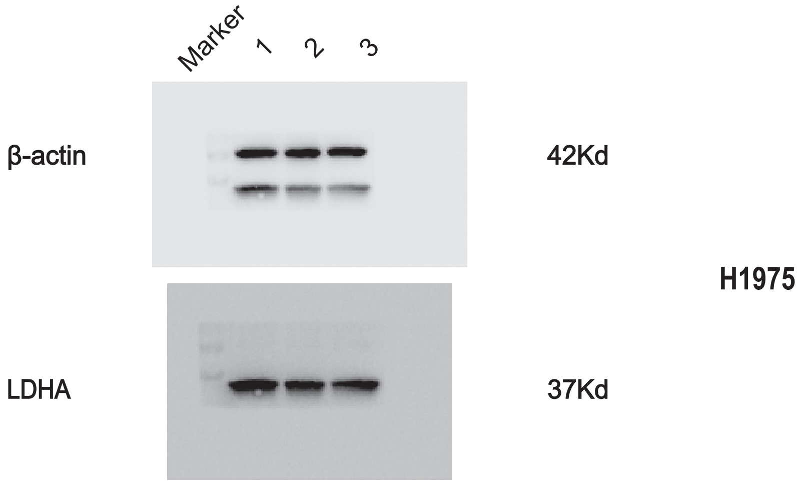

Figure 3B(H1975). Fluorescent imaging. 1. Con, 2. 10nM, 3. 20nM(Oxmate)

Fig 7B

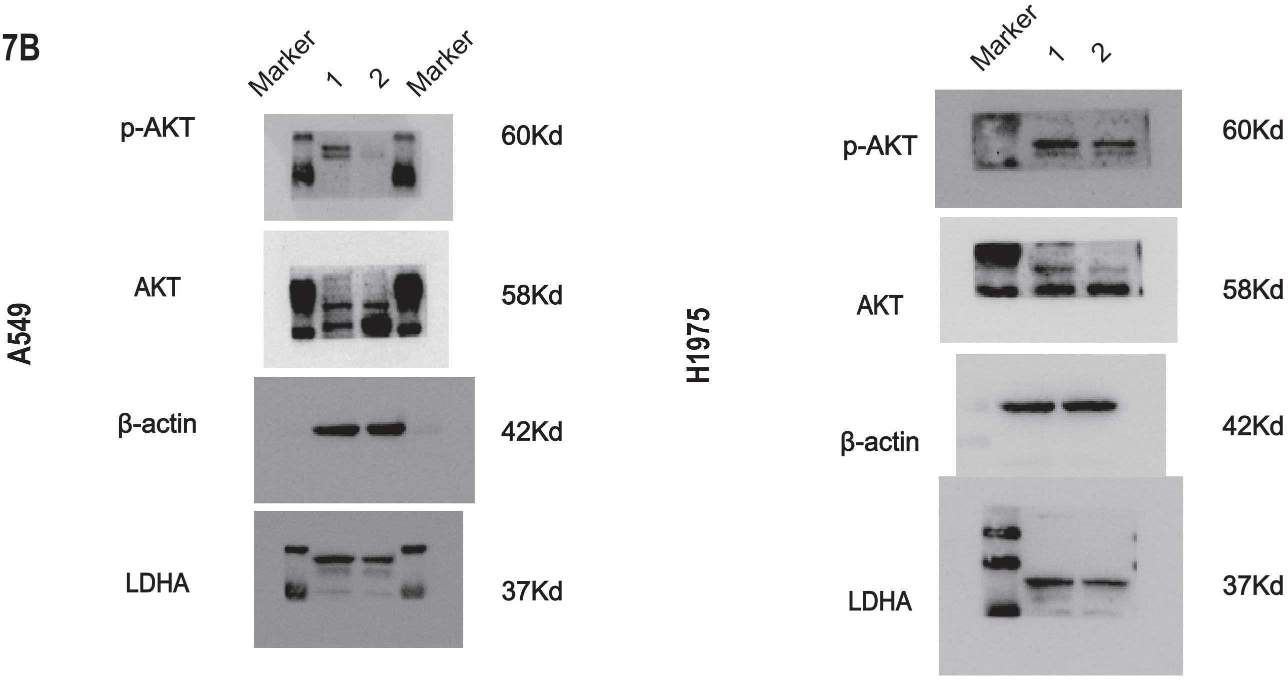

Figure 7B． Fluorescent imaging. 1. siNC, 2. si,

Fig 7D

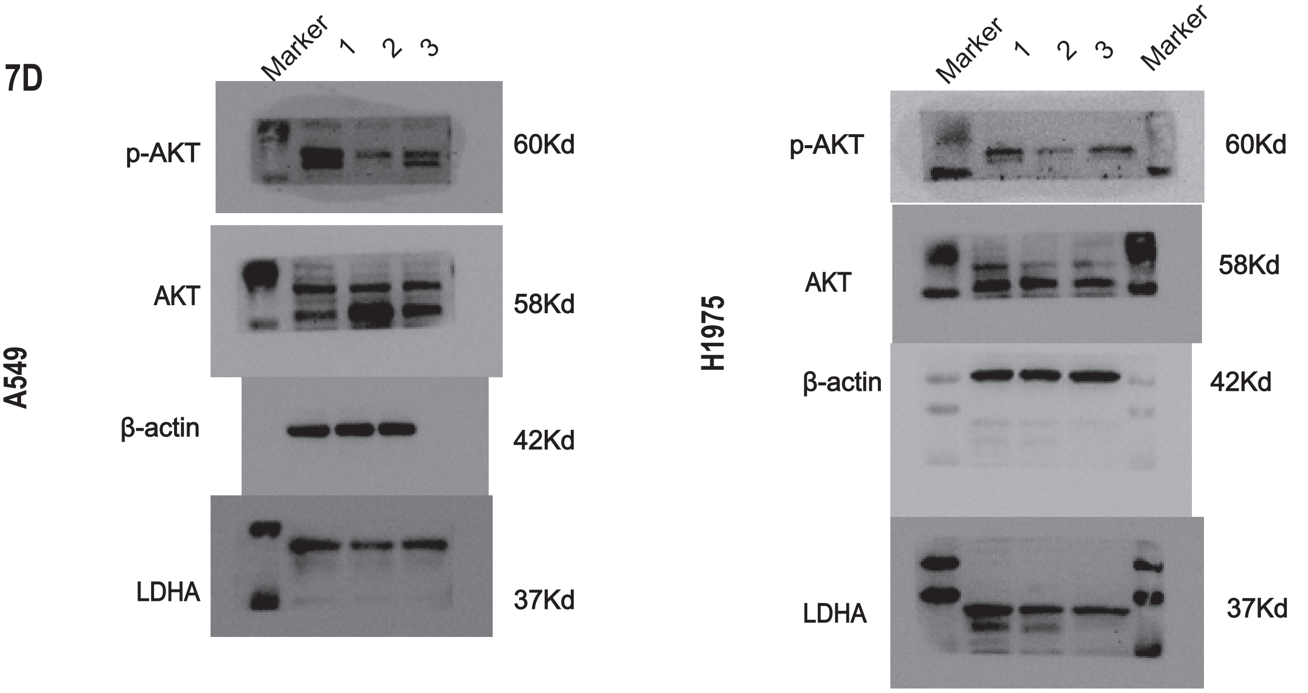

Figure 7D． Fluorescent imaging. 1. siNC, 2. si, 3. si+NAD

Fig 7E

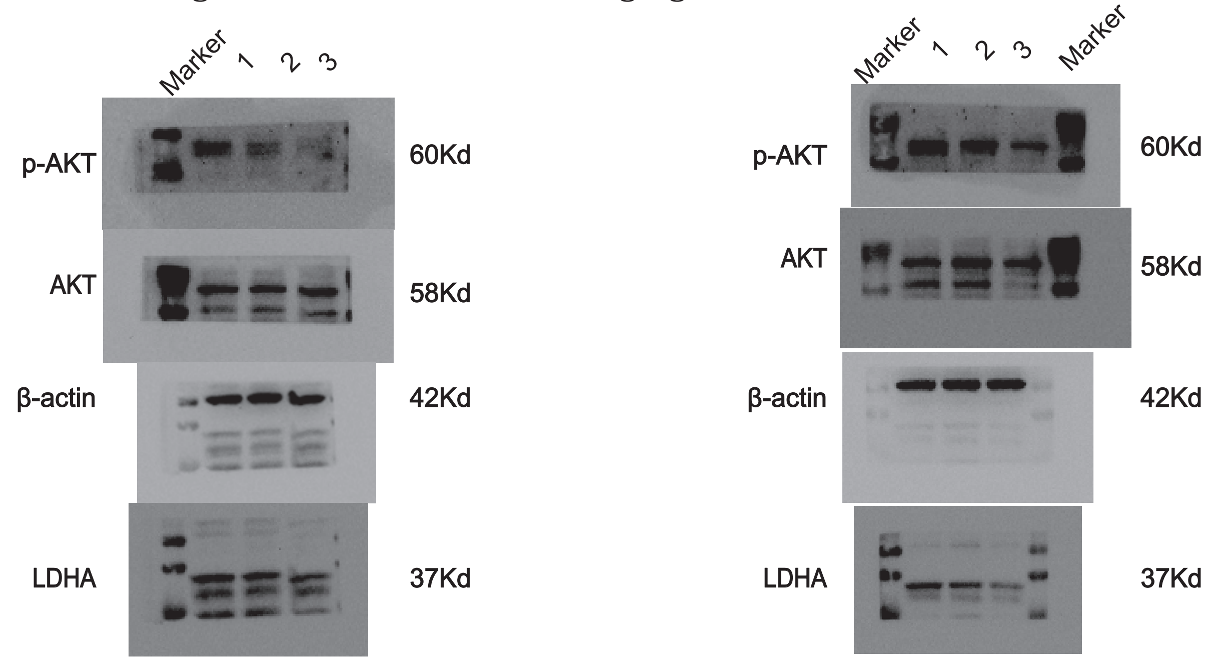

Figure 7E． Fluorescent imaging. 1. con, 2. 5uM, 3. 10uM(LY294002)
